# Collagen Nanoparticle-Mediated Brain Silymarin Delivery: An Approach for Treating Cerebral Ischemia and Reperfusion-Induced Brain Injury

**DOI:** 10.3389/fnins.2020.538404

**Published:** 2020-10-26

**Authors:** Pankaj Rathore, Indu Arora, Shweta Rastogi, Mohd Akhtar, Shruti Singh, Mohammed Samim

**Affiliations:** ^1^Department of Chemistry, School of Chemical & Life Sciences, Jamia Hamdard, New Delhi, India; ^2^Department of Biomedical Sciences, Shaheed Rajguru College, University of Delhi, New Delhi, India; ^3^Department of Chemistry, Hansraj College, University of Delhi, New Delhi, India; ^4^Department of Pharmacology, School of Pharmaceutical Education & Research, Jamia Hamdard, New Delhi, India; ^5^Department of Botany, School of Chemical & Life Sciences, Jamia Hamdard, New Delhi, India

**Keywords:** ischemia, reperfusion, apoptosis, stroke, neuroprotection

## Abstract

Silymarin is a bioactive constituent isolated from milk thistle (*Silybum marinum*). Since its discovery, silymarin has been considered a gold standard drug in treating ailments related to the liver, resulting from alcohol consumption and viral hepatitis. This hepatoprotective nature of silymarin arises out of antioxidative and tissue-regenerating properties of silymarin. However, several recent studies have established the neuroprotective link of silymarin, too. Thus, the current investigation was aimed at exploring the neuroprotective effect of nanosilymarin (silymarin encapsulated inside collagen-based polymeric nanoparticulate drug delivery system). The study aimed at bringing out the role of nanoparticles in enhancing the therapeutic effect of silymarin against neuronal injury, originating out of oxidative-stress-related brain damages in focal cerebral ischemia. Collagen-based micellar nanoparticles were prepared and stabilized using 3-ethyl carbodiimide-hydrochloride (EDC-Hcl) and malondialdehyde (MDA) as crosslinkers. Nanoparticles were characterized using dynamic light scattering (DLS), transmission electron microscopy (TEM), and Fourier transform infrared (FT-IR) spectroscopy techniques, and the size of nanoparticles was found to be around 48 nm. Male albino Wistar rats were pretreated with three different doses of nanosilymarin of 10, 100, and 1,000 μg/kg b.wt and a dose of free silymarin of 100 mg/kg b.wt intraperitoneally (i.p.) for 7 days. Focal cerebral ischemia was induced using the middle cerebral artery occlusion (MCAO) model on the eighth day for 1 h followed by 24 h reperfusion. The animals were then evaluated for neurobehavioral, infarct analysis, biochemical, histopathological, and immunohistochemical studies. All the above parameters showed remarkable improvement in nanosilymarin-treated groups in comparison to the silymarin-treated group. Nanoparticle encapsulation of drug enhanced neuroprotection by increasing drug bioavailability and targeting. Thus, the present study concluded with satisfactory results, showing the critical role played by nanoparticles in improving the neuroprotection at very low drug doses.

## Introduction

Stroke has become the second common cause of deaths worldwide at 11.8%, after deaths due to ischemic heart disease at 14.8% (Feigin et al., 2017). In addition, stroke is the third largest neurological disorder, which causes disability along with mortality and morbidity (Ahmad et al., 2013, 2016a,b). Cerebral stroke causes a deficiency of oxygen and glucose due to blockage of blood flow to a region of the brain (Zemke et al., 2004), resulting in damage to the physiological activity of the brain. Ischemia/reperfusion (I/R)-based injury results in cellular damage by triggering a series of events such as the generation of free radicals, excitotoxicity, inflammatory reactions, and apoptosis, affecting the proper functioning of the brain (Rama and García, 2016). The brain damage associated with ischemia might be due to metabolic stress suffered by the neurons, which do not store adenosine triphosphate (ATP) and rely on oxidative metabolism. This results in a lack of proper functioning of the mitochondria, thus disturbing the delicate redox balance, leading to a series of events following the ischemic cascade resulting in the combined effect of necrotic and apoptotic cell death pathways (Chauhan et al., 2017). Reactive oxygen species (ROS) generated during oxidative stress destroy cellular integrity by mediating damage to various cell components like lipids, proteins, membranes, and dinucleotide acid (DNA) (Thiyagarajan and Sharma, 2004; Rodrigo et al., 2005; Mukherjee et al., 2007). Oxidative injury due to ischemic damage becomes intense in those areas where blood supply has been restored, as a reflow of blood into the brain regions increases the level of oxygen. Apoptosis plays a significant role in neuronal death during ischemia (Liu et al., 2014). Caspases, which are involved in three major mechanisms−mitochondrial pathways, endoplasmic stress, and death receptors that account for apoptosis−have a crucial role to play in apoptotic cell death. The MCAO model was explicitly chosen from among all the animal models of ischemic stroke, as it closely imitates stroke in humans (Fluri et al., 2015). Treatment strategies for treating various central nervous system (CNS) disorders require crossing specific barriers possessed by CNS for protecting itself from invading pathogens and neurotoxic molecules. These barriers are essential interfaces between CNS and periphery. Blood–brain barrier (BBB) is one such barrier whose protective features control ion and molecule movement and protect brain homeostasis. The selective permeability to the flow of molecules across BBB has posed the biggest challenge to brain drug delivery. This not only reduces drug efficacy but also requires the drug to be administered in high doses to achieve desired effects resulting in undesirable side effects.

The last few decades have seen a surge in the use of drugs like silymarin in treating CNS diseases due to its neuroprotective effect. Silymarin, a well-known polyphenolic flavonoid belonging to the Asteraceae family, is isolated from fruits and seeds of milk thistle and has found use in treating various hepatic disorders (El-Kamary et al., 2009). Silymarin has been reported to show antioxidant activity in the treatment of multiple neurodegenerative diseases like stroke, Parkinson’s disease, and aging (Galhardi et al., 2009; Muley et al., 2012). It is also known to have anti-inflammatory (Hou et al., 2010), antiapoptotic (Karimi et al., 2011), and anticancer properties (Chen et al., 2009). The antioxidative property can be attributed to free radical scavenging (Jain et al., 2012) along with activation of antioxidant defense mechanisms like increased level of glutathione (GSH) content and enhanced superoxide dismutase (SOD) activity (Surai, 2015). Silymarin has also been known to reduce the extent of lipid peroxidation (Surai, 2015). Despite having so many curative properties, the use of silymarin has been hampered by its poor bioavailability due to low aqueous solubility, low intestinal absorption, rapid metabolism, and rapid excretion in bile and urine. Thus, there is a need to incorporate silymarin into a form that can augment its bioavailability. Presently, treatment strategies being developed against stroke are aimed at providing neuroprotection after ischemic injury, but its efficacy might reduce due to delayed initiation of therapy. Thus, pretreatment with a neuroprotective drug before the onset of ischemia could offer definite advantages in patients at high risk of getting an ischemic stroke. Moreover, patients who have already suffered a mild initial stroke are at higher risk for recurrence, making it necessary to consider secondary pretreatment as long-term neuroprotection for them (Zhao and Rempe, 2011). The importance of supplementary preventive drugs in reducing neuronal damage after ischemic injury makes the pretreatment approach even more crucial (Puig et al., 2018). It has been shown that several drugs can have a beneficial effect against stroke by decreasing oxidative stress and inflammation (Hong and Lee, 2015). The current study was designed, keeping in mind the clinical significance of pretreatment and benefits associated with herbal therapeutic interventions like broad therapeutic range and lesser known side effects. Moreover, the use of polymeric micelles as drug delivery systems has gained momentum owing to the distinct advantages associated with them like high drug loading capacity, preventing drug degradation and sustained drug release (Elzoghby et al., 2016). In the current study, silymarin-encapsulated collagen nanoparticles have been prepared by using a simple but efficient method for brain-targeted drug delivery. Biopolymer collagen was strategically chosen, as it is biocompatible, biodegradable, and weakly antigenic (Chan et al., 2020), and Tween^©^80 aids in drug transport across the BBB by imitating through low-density lipoprotein (LDL) receptors present on the brain surface (Sun et al., 2004; Aboutaleb et al., 2014). The micellar structure was formed by Tween^©^80, a polyethylene sorbitol ester, which is a non-ionic surfactant. Micelle formation begins by spherical aggregation of polysorbate, in which the hydrocarbon chains forming the hydrophobic part point towards the core and the ethylene oxide subunits comprising the hydrophilic portion point towards the periphery. Silymarin being hydrophobic stabilizes the inner region of the nanoparticle by van der Waals interactions, and the hydrophilic periphery is stabilized by hydrogen bond formation with the surroundings. The collagen nanoparticle was further stabilized by crosslinking using EDC-Hcl and MDA. The novelty of the synthesized collagen nanoparticle lies in its design, which makes it an efficient brain-targeted drug delivery system. The use of collagen not only weakens immunogenic response but also makes the surface hydrophilic; this helps the nanoparticle escape the reticuloendothelial system (RES), thus imparting it stealth properties. The use of Tween^©^80 also helps reduce RES uptake in addition to brain targeting. Furthermore, crosslinking collagen not only stabilizes the nanoparticle but also aids in drug entrapment, thus helping achieve slow and sustained drug release for optimum therapeutic effect.

In the current investigation, we have hypothesized that pretreatment with silymarin-encapsulated collagen nanoparticles offered a better approach to reduce I/R injury post-MCAO-induced cerebral ischemia by providing better neuroprotection.

## Materials and Methods

### Chemicals and Reagents

Chemicals acquired from SRL Chemicals include glutathione (GSH), oxidized glutathione (GSSG), glutathione reductase (GR), reduced nicotinamide adenine dinucleotide phosphate (NADPH), 1,2-dithio-bis-nitrobenzoic acid (DTNB), 1-chloro-2,4-dinitrobenzene (CDNB), trichloroacetic acid (TCA), sodium hydroxide (NaOH), copper sulfate (CuSO_4_), ethylenediaminetetraacetic acid (EDTA), disodium orthophosphate (Na_2_HPO_4_), sodium dihydrogen phosphate (NaH_2_PO_4_), sodium potassium tartrate, Folin–Ciocalteu reagent (FCR) sodium carboxymethyl cellulose, and sodium carbonate (Na_2_CO_3_). Chemicals acquired from Thomas Baker Chemicals include hydrogen peroxide (H_2_O_2_) and sodium azide (NaN_3_). Chemicals acquired from Spectrochem Chemicals include thiobarbituric acid (TBA) and 1-(3-dimethylaminopropyl)-3-ethyl carbodiimide-hydrochloride (EDC-Hcl). Chemicals acquired from SD Fine Chemicals include Tween^©^80 and epinephrine (−). Chemicals acquired from Hi Media Chemicals include bovine serum albumin (BSA) and 5-sulfosalicylic acid. Chemicals acquired from Sigma-Aldrich include silymarin, 2,3,5-triphenyl tetrazolium chloride (TTC), MDA, Dulbecco’s modified Eagle’s medium (DMEM), 3-(4,5-dimethylthiazol-2-yl)-2,5-diphenyl tetrazolium bromide (MTT), fetal bovine serum (FBS), and finally, Ovicoll Collagen was acquired from Holista Colltech Limited Australia.

### Synthesis of Collagen Nanoparticles

**FIGURE 1:**
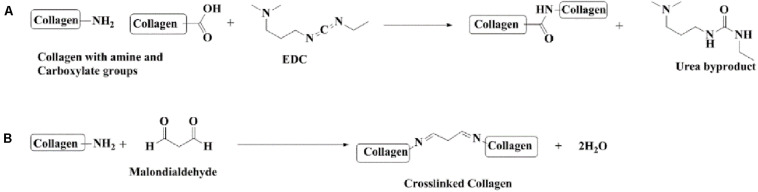
Chemical reactions involved in the preparation of collagen nanoparticles. **(A)** Cross-linking collagen using EDC-Hcl. **(B)** Cross-linking collagen using MDA.

Forty milliliters of double distilled water was kept on stirrer at a temperature of 8°C, and 1 ml of Tween^©^80 was added to it. After 4 h, a solution containing 6 mg collagen dissolved in 10 ml of double distilled water was added dropwise into the solution kept on the stirrer. This was followed by the addition of 3 mg EDC-Hcl after 2 h and consecutively adding 3 mg of MDA after 2 h. The final solution was kept on the stirrer overnight. The dialysis of finally prepared nanoparticle solution was done for 48 h using a spectropore membrane dialysis bag (celluSep^®^, 12 kD cutoff); the dialysis process involved changing the medium distilled water after every 4 h (Figure 1).

### Characterization of Collagen Nanoparticles

#### Drug Loading

Silymarin was dissolved in dimethyl sulfoxide (DMSO) (5 mg/ml), and the solution of the drug was added slowly to an aqueous solution of nanoparticles with continuous vortexing and sonication until the solution began to start losing its transparency. At this point, further addition of drug solution was stopped to avoid an excess of drug getting entrapped into the nanoparticles. Silymarin, being hydrophobic, goes into the hydrophobic core of the collagen nanoparticles. The drug-encapsulated collagen nanoparticle solution was finally dialyzed for 24 h with a constant change of water after every 2 h to remove any dissolved DMSO. After completion of dialysis, the drug-encapsulated nanoparticle solution was lyophilized and stored for further usage.

#### Physicochemical Characterization

FT-IR spectra was obtained by using an FT-IR spectrophotometer (Bruker Alpha Instrument). Using FT-IR spectra, functional groups present can be detected. It was performed to detect if any crosslinking has taken place in the collagen nanoparticle after adding EDC-Hcl and MDA. FT-IR spectrum was recorded in the frequency range of 4,000 to 400 cm^–1^. Particle size (PS) and polydispersity index (PDI) were determined using dynamic light scattering (DLS) technique on a Zetasizer Nano ZS (Malvern Instruments Corp., Malvern, United Kingdom). All samples were diluted with Millipore-filtered deionized water to an appropriate scattering intensity. The nanoformulation was morphologically examined using a technique called transmission electron microscopy (TEM) (TALOS Instrument, Thermo Fischer Scientific, United States) at AIIMS, New Delhi. The sample preparation involved putting a few drops of nanoparticles on a carbon-coated copper grid and was negatively stained using 1% phosphotungstic acid (PTA). Excess of stain solution was removed using a filter paper followed by air drying. The next step required the examination of stained films using a TEM instrument.

#### Nanoparticle Encapsulation Efficiency (EE%)

It is a measure of the percentage of drug that has been encapsulated inside the nanoparticles relative to the total drug that has been added. It specifies the quantity of the drug that has been entrapped inside the nanoparticles and the drug that remains unentrapped is present in the dispersion medium. The encapsulation efficiency (EE%) of silymarin-loaded nanoparticles were obtained by centrifuging the drug-loaded nanoformulation for 30 min at 14,000 rpm, maintaining the temperature at 4°C, and then, the amount of unentrapped (free) silymarin was measured by obtaining the absorbance of the supernatant at 288 nm. EE% was determined as follows:

EE%=[( mass of total drug−mass of the freedrug)/mml:mass of totaldrug]×100

where the mass of total drug denotes the theoretical amount of silymarin in the NPs, and the mass of free drug is the amount of silymarin practically measured in the supernatant. Experiments were run in triplicates.

The loading efficiency (LE%) denotes the amount of drug associated with the unit weight of nanoparticles. LE% can be determined as follows:

LE%=[(mml:mass of total drug− mml:mass of the freedrug)/(weight of nanoparticles)]×100

#### *In vitro* Drug Release Study

The *in vitro* release kinetics study of a drug from its nanoformulation is necessary for the development of an effective nanocarrier. This study involved analyzing the *in vitro* release kinetics of silymarin from collagen nanoparticles by the dialysis bag method. Briefly, about 100 mg lyophilized powder of silymarin-loaded collagen nanoparticles was dissolved in 1 ml of distilled water. The above solution was kept for dialysis in a beaker containing 50 ml of phosphate buffer solution (pH 7.4, including 1% Tween^©^80), which was used as a receptor medium, as it provided proper sink conditions due to the low aqueous solubility of silymarin. The biological conditions were simulated by carrying out the whole process in an incubator maintained at 37°C. The drug slowly releases into the sink medium. The amount of drug released was measured by taking out about 2 ml of solution from the sink medium and immediately recording the absorbance at 288 nm. The volume of the sink medium was compensated by simultaneously adding an equal amount of the fresh corresponding medium. Similarly, the release kinetics of free silymarin dissolved in DMSO was determined to maintain similar conditions. The standard absorbance curve of silymarin was used to determine the amount of drug present in the solution withdrawn at different time intervals.

The percentage of silymarin released at different time intervals was calculated as follows:

$$\text{Release}\left(\%\right)=\left\{{\left[\text{Drug}\right]}_{\text{released}}/{\left[\text{Drug}\right]}_{\text{total}}\right\}\times 100$$

where [Drug]_released_ depicts the concentration of drug released at time t, and [Drug]_total_ represents the concentration of total drug that has got trapped inside the collagen nanoparticles. The data were presented as mean ± SD (*n* = 5).

#### Cell Viability Assay

Cell viability was assessed by performing 3-(4,5-dimethylthiazol-2-yl)-2,5-diphenyltetrazolium bromide (MTT) assay using human umbilical vein endothelial cells (HUVECs) obtained from the National Centre for Cell Science (NCCS), Pune, India. The cells were cultured in DMEM medium with 10% FBS, and the medium was supplemented with antibiotics. The cells were seeded at 1 × 10^5^ cells/well in 96-well plates and incubated for 24 h at 37°C in a 5% CO_2_ incubator. Thereafter, treatment with different concentrations of blank nanoparticles (NPs), silymarin-loaded NPs, and silymarin in the concentration range of 0.025–200 μg/ml was performed, and cells were kept for 24 h incubation. Following incubation, the test samples were removed, 20 μl MTT reagent (5 mg/ml stock solution) was added, and plates were further incubated for 4 h at 37°C. The formed formazan crystals were dissolved in 100 μl DMSO, and the absorbance was measured at 570 nm using a microplate spectrophotometer (Powerwave XS2, Bio Tek Instruments, United States) (Vakili Zahir et al., 2018). The resulting absorbance values were used for calculating the percentage viability of the cells. The experiments were performed in triplicates, and results were expressed as percentage cell viability with untreated cells as control.

### *In vivo* Studies

#### Animal Ethics

Male albino Wistar rats having weights between 250 and 300 g were procured (ethical review under approval no. 966) from the Central Animal House facility (CAHF) at Jamia Hamdard, New Delhi, India with registration no. 173/GO/Re/2000/CPCSEA. All the animals were kept inside polypropylene cages, each having four animals. The cages were housed in air-conditioned rooms maintained at 22–25°C with exposure to a 12-h light/dark cycles. The animals were given access to a standard pellet diet and water *ad libitum*. All the animals were put to prior acclimatization for 1 week so that they can adapt to their environment before starting the experiment. The diet was withdrawn 12 h before the surgical procedure. The experimental protocols were reviewed and approved according to guidelines set by the Animal Ethics Committee CPCSEA under the Government of India. The protocol was approved by IAEC Jamia Hamdard, New Delhi, India.

#### Dose Selection and Drug Administration

For studying the neuroprotective effects of nanosilymarin, all animals were divided mainly into six groups, with each group comprising of six animals (*n* = 6), and dosing schedule was as follows:

Group I−served as control (C) and animals of this group were administered with void collagen nanoparticles intraperitoneally (i.p.) for 7 days.

Group II−served as ischemic (I).

Group III−given nanosilymarin (NS1-10 μg/kg b.wt) i.p. for 7 days.

Group IV−given nanosilymarin (NS2-100 μg/kg b.wt) i.p. for 7 days.

Group V−given nanosilymarin (NS3–1,000 μg/kg b.wt) i.p. for 7 days.

Group VI−given free silymarin (100 mg/kg b.wt) by dissolving in 0.3% w/v carboxymethyl cellulose i.p. for 7 days.

In group I animals, all the surgical procedures were carried out, but the middle cerebral artery (MCA) was not occluded. All the animals of groups II–VI underwent MCAO for 1 h on the eighth day, followed by reperfusion for 24 h. The animals recovered from anesthesia within 4–5 h of suture withdrawal after MCAO. After a reperfusion period of 24 h, all the animals were put to neurobehavioral assessment. Then, the animals were sacrificed after decapitation followed by removal of brains for performing various *in vivo* studies like infarct analysis, biochemical estimations, and histopathological and immunohistochemical studies.

#### Ischemia Induction and Reperfusion

The MCAO was carried out by using the intraluminal filament model. In brief, animals were anesthetized using chloral hydrate (400 mg/kg b.wt, i.p.). The external carotid artery (ECA) was exposed from the ventral side by making an incision of 1 cm on the neck skin. Then, polylysine-coated monofilament (4 0-3033REPK10, Doccol Corporation, United States) with a flexible and smooth tip was introduced into the ECA and carefully advanced into the MCA through the internal carotid artery (ICA) for about 17–20 mm length until a slight hindrance was felt; this indicates that the filament has moved beyond the proximal part of the anterior cerebral artery (ACA). At the point of the obstruction, the origin of MCA has been blocked by intraluminal suture, thus obstructing all sources of blood flow from the posterior cerebral artery (PCA), ICA, and ACA. An hour after induction of ischemia, the intraluminal filament was slowly withdrawn, and animals were put back into their cages for 24 h reperfusion. All the procedures were the same in the control group, but no MCA occlusion was performed. After surgery, all the animals were kept in individual cages in well-ventilated rooms at room temperature until they gained full consciousness. The animals were given easy access to food and water to avoid any kind of physical exertion.

#### Neurobehavioral Assessment

##### Rota-rod test

After 24 h reperfusion, the motor coordination of all experimental animals was evaluated using the rota-rod apparatus Omni rotor (Omnitech electronics Inc., Columbus, United States) using the previously described method (Kelly et al., 1998). The experimental setup comprised of a rotating rod of 75 mm diameter, which was divided into four sections for testing four animals at a time. The speed of the rotating shaft was set at 10 cycles per minute with a cutoff time of 180 s. The time for which animals could stay on the rotating rod (latency time) was recorded for three trials for each animal at 5-min intervals. The mean of three trials was presented in the data. The apparatus automatically recorded time within 0.1 s of rat falling off the rotating shaft. All the animals were given behavioral training five times a day (interval: 1 h) for 5 days before MCAO induction until they could walk on the rotating rod for at least 10 s.

##### Spontaneous motor activity

Spontaneous motor activity (SMA) analysis was performed after 24 h of reperfusion period to assess the neurological deficit using the previously described method (Raza et al., 2011b). SMA was evaluated by observing the activity of animals in their cages for 5 min. Neurological scoring was given as follows: 0 = rat freely moved around exploring the environment of the cage; 1 = rat moved inside the cage but showed hesitation in approaching all the sides of the cage; 2 = rat hardly moved inside the cage and exhibited postural abnormalities bent toward paretic side; 3 = rat did not show any movement at all and remained static with posture bent toward the paretic side.

##### Flexion test

Flexion test (FT) was done to assess the neurological deficit using the previously described method (Vaibhav et al., 2012). Accordingly, scoring was done based on four scales: 0 = no neurological deficit was observed; 1 = showed contralateral forelimb flexion with wrist flexion and shoulder adduction; 2 = showed a reduction in the ability to resist the lateral push; and 3 = showed circular movements toward the paretic side.

##### Grip strength test

Grip strength test was evaluated using the previously described method (Moran et al., 1995). A 50-cm long string was tied between two vertical supports, and evaluation of animal activity was done based on the following scales: **0** = rat fall down; **1** = rat kept hanging by gripping the string with two forepaws; **2** = in addition to grasping the string with forepaws rat also attempted climbing the string; **3** = rat kept hanging onto the string using both forepaws along with one or both the hind limbs; **4** = rat kept hanging onto the string using both forepaws with the tail enfolded around the string; and **5** = rat escaped. Three successive trials were conducted for each animal, and the highest score was taken as the best score for that animal. All the test animals were given 5 days of training before MCAO induction. Each animal was made to hang onto a string in the experimental setup, three trials per day at an interval of 1 h for at least 5 s.

#### Measurement of Infarct Size

In this investigation, 24 h after dosing ended, all the animals were sacrificed by cervical dislocation. The measurement of infarct volume was made by using TTC staining method (Liu et al., 2016). TTC itself is colorless, but succinate dehydrogenase reduces it into deep red colored formazan (Sanchez-Bezanilla et al., 2019). Areas of the brain where there was necrosis effect lacked dehydrogenase activity and showed white or pale yellow color, as there was no staining. After sacrifice, brains were surgically removed and hind region removed before placing the brain in a brain matrix (Zivic Instruments). In the brain matrix, about 3-mm thick coronal sections of the brain were sliced out and incubated at 37°C in a solution of TTC (0.1% w/v) in PBS to assess cerebral infarct size. The digital images of each brain section was captured using a digital camera, and infarct volume was measured by analyzing the images using NIH Image J analysis software (version 1.52a). The infarct volume was corrected for error due to edema, and corrected infarct volume was calculated as previously described (Kim et al., 2011). The corrected infarct area for each brain section was calculated as follows:

$$\begin{array}{l} \;{\text{ Corrected infarct area (mm}}^{2})=\\ \text{total area of contralateral hemisphere}-\\ \text{intact (non}-\text{infarct) area of the ipsilateral hemisphere.} \end{array}$$

The total infarct volume (mm^3^) of each brain section was calculated by multiplying the above calculated area with the thickness of each section (3 mm). The final total infarct volume of the whole brain was calculated by summing up the infarct volume of each of the five coronal sections. The percentage of infarct volume was calculated as follows:

Infarct volumml:me(%)=(total volumml:me of infarct/volume of whole brain)×100

#### Biochemical Studies

##### Tissue preparation

All the experimental animals were sacrificed, and their brains were carefully dissected to take out cortex section. The tissue for homogenization at 10% (w/v) was prepared in 10 mmol/L PBS (pH 7.4). Then, its centrifugation was done at 800 × *g* for 5 min at 4°C to get supernatant (S1). A portion of the above-prepared supernatant (S1) was used for doing the thiobarbituric acid reactive substances (TBARS) assay, while the remaining supernatant was centrifuged at 10,500 × *g* for 15 min at 4°C. The supernatant finally obtained after centrifuging for the second time was used for estimating the level of glutathione (GSH) followed by the activity of various antioxidant enzymes like glutathione-S-transferase (GST), glutathione reductase (GR), glutathione peroxidase (GP_X_), superoxide dismutase (SOD), and catalase (CAT).

##### Determination of lipid peroxidation

Lipid peroxidation (LPO) estimation was done by performing the TBARS assay, which forms as a by-product of lipid peroxidation. Estimation of TBARS was done using the previously described method (Utley et al., 1967) with some modifications. Homogenate (0.1 ml) was incubated at 37°C using a metabolic water bath shaker. Another 0.1 ml of the same homogenate was incubated at 0°C. Both the homogenates were incubated for 1 h, followed by the addition of 0.5 ml 0.67% (w/v) TBA and 0.5 ml 10% (w/v) TCA to both the above homogenates. Then, both the homogenate samples were centrifuged at 3,000 × *g* for 15 min. The supernatant obtained after centrifugation of the above samples was collected in two separate test tubes and kept in a boiling water bath for 10 min. The absorbance of solutions in both the test tubes was taken at 535 nm at room temperature. The rate of lipid peroxidation is measured as μmole of TBARS formed/h/g tissue using a molar extinction coefficient of 1.56 × 10^5^ M^–1^ cm^–1^.

##### Estimation of reduced glutathione content

The GSH content was determined using the previously described method (Jollow et al., 1974). One milliliter of postmitochondrial supernatant (PMS) fraction (10%) was added to 1.0 ml of 4% (w/v) sulfosalicylic acid, and the mixture was incubated at 4°C for 1 h, followed by centrifugation at 1,200 × *g* and temperature of 4°C for 15 min. In a sample vial, 0.4 ml of the above filtered aliquot was taken, and 2.2 ml phosphate buffer (0.1 M, pH 7.4) and 0.4 ml DTNB (10 mM) were added to make the total volume to 3.0 ml. The absorbance of the above formed yellow mixture was immediately measured at 412 nm. The GSH levels were reported as μmol of DTNB conjugate formed/g tissue using a molar extinction coefficient of 13.6 × 10^3^ M^–1^ cm^–1^.

##### Measurement of glutathione reductase activity

The GR activity was determined using the previously described method (Carlberg and Mannervik, 1975) with slight modifications as described (Mohandas et al., 1984). The assay mixture consisted of 0.1 ml 10% PMS, 1.65 ml PB (0.1 M, pH 7.6), 0.1 ml NADPH (0.1 mM), 0.05 ml GSSG (1.0 mM), and 0.1 ml EDTA (0.5 mM), making the total volume to 2 ml. The enzyme activity was measured as the disappearance of NADPH at 340 nm and was reported as nmol of NADPH oxidized/min/mg protein using a molar extinction coefficient of 6.22 × 10^3^ M^–1^ cm^–1^.

##### Measurement of glutathione peroxidase activity

The GPx activity was determined using the previously described method (Mohandas et al., 1984). The reaction mixture comprised of 1.44 ml PB (0.1 M, pH 7.4), 0.1 ml sodium azide (1.0 mM), 0.1 ml EDTA (1.0 mM), 0.05 ml GR (1 IU/ml), 0.05 ml reduced glutathione (1 mM), 0.1 ml NADPH (0.2 mM), 0.01 ml H_2_O_2_ (0.25 mM), and 0.1 ml 10% PMS, making the total volume to 2.0 ml. The disappearance of NADPH was measured by taking absorbance at 340 nm at room temperature. The enzyme activity was expressed as μmol of NADPH oxidized/min/mg protein using a molar extinction coefficient of 6.22 × 10^3^ M^–1^ cm^–1^.

##### Measurement of superoxide dismutase activity

Superoxide dismutase activity was determined using the previously described method (Stevens et al., 2000). The reaction mixture included 1.8 ml glycine buffer (50 mM, pH 10.4) and 0.2 ml PMS. The reaction started after adding (−) epinephrine. The enzyme activity was measured by monitoring the auto-oxidation of (−) epinephrine for 3 min at a pH of 10.4 at 480 nm. The enzyme activity was expressed as nmol (−) epinephrine protected from autooxidation/min/mg protein using a molar extinction coefficient of 4.02 × 10^3^ M^–1^ cm^–1^.

##### Measurement of catalase activity

The catalase activity was determined using the previously described method (Claiborne, 1985). The reaction mixture included 2.0 ml PB (0.1 M, pH 7.4), 0.95 ml hydrogen peroxide (0.019 M), and 0.05 ml PMS (10%), making the total volume to 3.0 ml. Enzyme activity was measured by monitoring the change in absorbance at 240 nm. The catalase activity was expressed as nmol of H_2_O_2_ consumed/min/mg protein using a molar extinction coefficient of 40 M^–1^ cm^–1^.

##### Measurement of glutathione-S-transferase Activity

The GST activity was determined using the previously described method (Habig et al., 1974). The reaction mixture comprised 2.4 ml PB (0.1 M, pH 6.5), 0.2 ml of reduced glutathione (1 mM), 0.2 ml CDNB (1 mM), and 0.2 ml PMS (10%), making the total volume to 3.0 ml. The enzyme activity was calculated by monitoring change in absorbance at 340 nm and was expressed as nmol of CDNB conjugate formed/min/mg protein using a molar extinction coefficient of 9.6 × 10^3^ M^–1^ cm^–1^.

##### Measurement of protein concentration

The protein concentration in all the samples was determined using the previously described method (Lowry et al., 1951). Peptide bonds present in protein makes a complex with alkaline copper sulfate reagent; this complex gives a blue color on adding Folin’s reagent. Briefly, 0.1 ml PMS (10% w/v) was diluted to 1 ml, and protein was precipitated by adding an equal volume of TCA (10% w/v). The samples were kept for overnight incubation at 4°C and later centrifuged at 800 × *g* for 5 min. The supernatant was discarded, and the pellets were dissolved in 5 ml of NaOH (1 N). Finally, 0.1 ml of aliquot was diluted to 1 ml with water, and then, 2.5 ml of alkaline copper sulfate reagent comprising Na_2_CO_3_ (2%), CuSO_4_ (1%), and sodium potassium tartrate (2%) was added to it. After adding alkaline CuSO_4_ reagent, the above mixture was kept for 10 min to allow complex formation followed by the addition of 0.25 ml of Folin–Ciocalteu reagent (FCR). In the end, a blue color developed after 30 min that was read at 660 nm. Bovine serum albumin (BSA, 0.1 mg/ml) was used as standard.

#### Histopathological Examination

After sacrificing rats, their brains were carefully dissected out, and the cortex region was separated and embedded in the paraffin. Coronal sections about 5 μm thick, having the cortex region, were serially sectioned using a microtome and examined under the microscope Olympus BX 51 after staining with hematoxylin and eosin to analyze any kind of pathological changes in the brain after ischemic injury.

#### Immunohistochemical Study

The immunostaining was performed following the procedure described in Thermo Scientific Ultra Vision ONE Large Volume Detection System Horseradish Peroxidase (HRP) Polymer (Ready-To-Use) kit (Shan et al., 1999; Karen et al., 2002). Dewaxing of paraffin sections was done using graded ethanol and dimethyl benzene. After induction of antigen retrieval by heat, the processing of incubated sections was done through 4% H_2_O_2_ for 15 min followed by application of ultra V block and incubation for 5 min at room temperature, as this would reduce non-specific background staining due to endogenous peroxidases. Brain sections were incubated with nuclear factor kappa B (NF-κB), inducible nitric oxide synthase (iNOS), and caspase-3 primary antibodies (Sigma Aldrich, United States) at room temperature for 1 h. Then, HRP polymer was applied, and incubation was done for 30 min at room temperature followed by washing with Tris-buffered saline (TBS) and further incubation with 3,3′-diaminobenzidine (DAB) for 5 min. Finally, hematoxylin was used for counterstaining; positive immunoreactivity in the cortex was definite in the deep-brown-stained cells. Finally, the slides were washed, dried, and mounted with dibutylphthalate polystyrene xylene (DPX) and analyzed using an Olympus BX 51 microscope. The captured images were further DAB quantified for assessing the expression of inflammatory and apoptotic markers by color deconvolution method using the public domain program NIH image J (Fiji version) software as described in Ruifrok (1997); Akinshipo et al. (2020) with slight modifications. The detailed experimental protocol has been provided in the Supplementary Material.

### Statistical Analysis

The data from individual groups were presented as the mean ± standard error of the mean (SEM) and standard deviation (SD). The analysis of differences between individual groups was done using ordinary one-way analysis of variance (ANOVA) followed by a *post hoc* Tukey’s Kramer multiple comparisons test using GraphPad Prism v8.2 (GraphPad Software, San Diego, CA, United States). The minimum criterion for statistical significance was set at *p* < 0.05 for all the comparisons.

## Results

### Physicochemical Characterization

#### FT-IR Analysis

**FIGURE 2:**
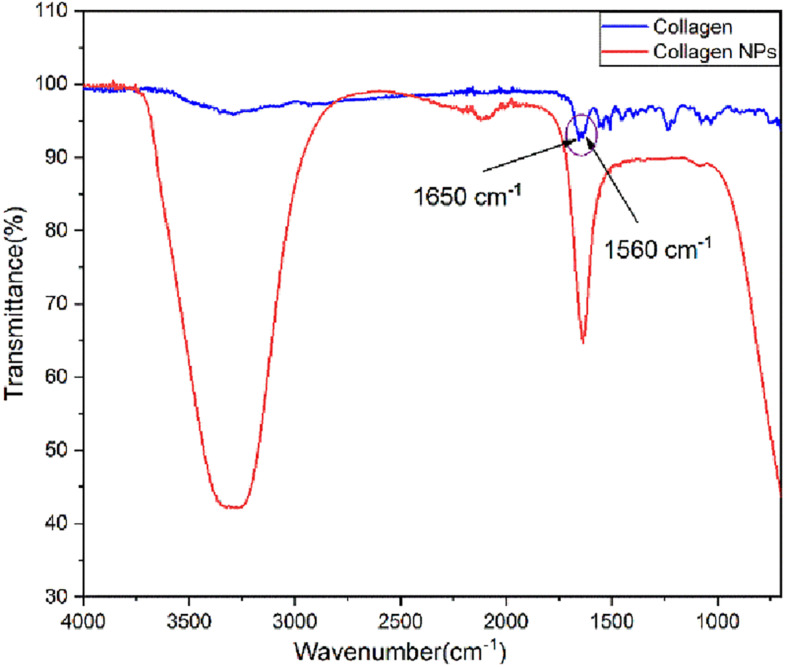
Fourier transform infrared (FT-IR) spectra of pure collagen and collagen nanoparticles.

Analysis of FT-IR spectra (Figure 2) shows that pure collagen shows the characteristic FT-IR absorption peak of amide I at ∼1,650 cm^–1^ reflecting the stretching vibration of the peptide carbonyl group (–CO) and an amide II peak at ∼1,560 cm^–1^ reflecting the –NH bending vibrations. After the addition of carboxyl-amine coupling reagent EDC-Hcl, it can be observed from the FT-IR spectra of collagen nanoparticles that –NH bending peak has disappeared. This indicates the formation of additional amide (–CONH) bonds, which is further confirmed by the appearance of a broad peak of –NH stretching between 3,100 and 3,500 cm^–1^. The peak for the carbonyl group (–CO) stretching is retained. In addition, the FT-IR spectra of collagen nanoparticles show that the intensity of peaks between 1,000 and 1,500 cm^–1^ has reduced, indicating the formation of amide bonds after the addition of EDC-Hcl and crosslinker MDA, thus further stabilizing collagen nanoparticles.

#### DLS and HR-TEM Analysis

**FIGURE 3:**
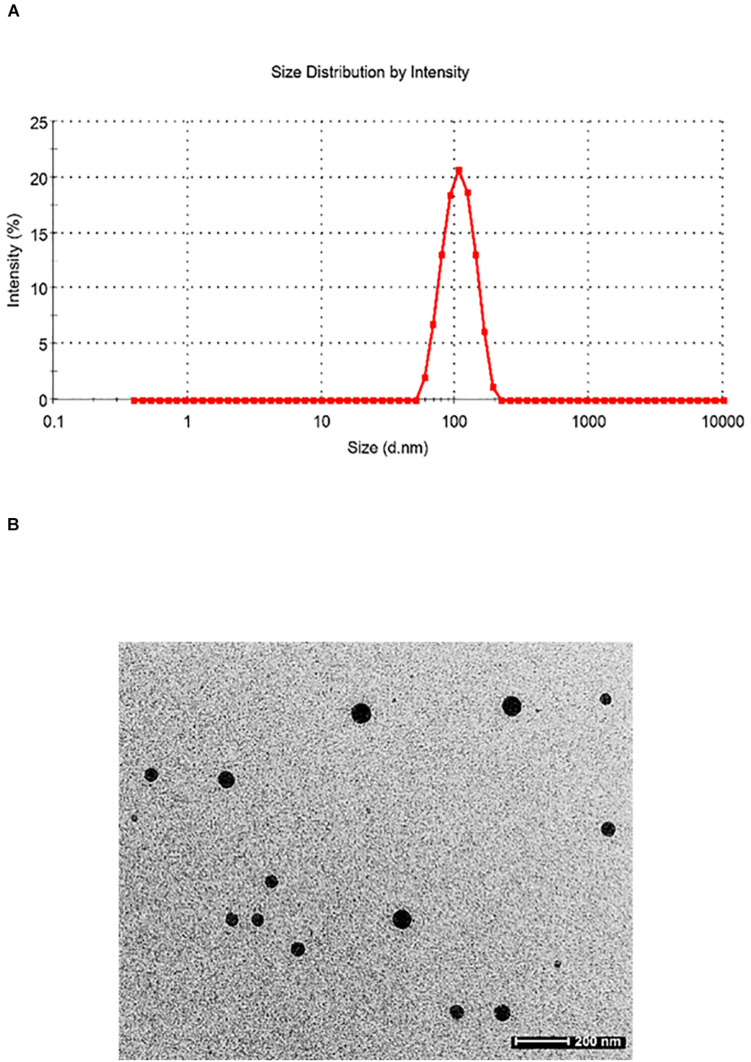
**(A)** Dynamic light scattering pattern of silymarin-encapsulated collagen nanoparticles. **(B)** Transmission electron microscopy of silymarin-encapsulated collagen nanoparticles.

Particle size plays a very crucial role in affecting the *in vivo* fate of NPs. Moreover, it determines the rate and extent of drug release. PDI measures the degree of heterogeneity of particles in a mixture with values in the range of 0–1, and values >0.5 indicate agglomeration of the particles. The nanoparticle size was analyzed using techniques like DLS and TEM. DLS of silymarin-loaded collagen nanoparticles showed an average hydrodynamic diameter of around 129.4 ± 16.4 nm (Figure 3A) and a PDI of 0.299 ± 0.024. The TEM analysis showed nanoparticles sized around 44.80 ± 3.83 nm with a spherical morphology (Figure 3B). The results were obtained for *n* = 3 data sets and represented as mean ± SD.

#### Nanoparticle Entrapment Efficiency (EE%)

The collagen nanoparticle entrapment efficiency EE% was found to be about 76.7 ± 2.4% with a loading efficiency of 3.17 ± 0.37% for *n* = 3 data sets and represented as mean ± SD.

#### *In vitro* Drug Release Study

**FIGURE 4:**
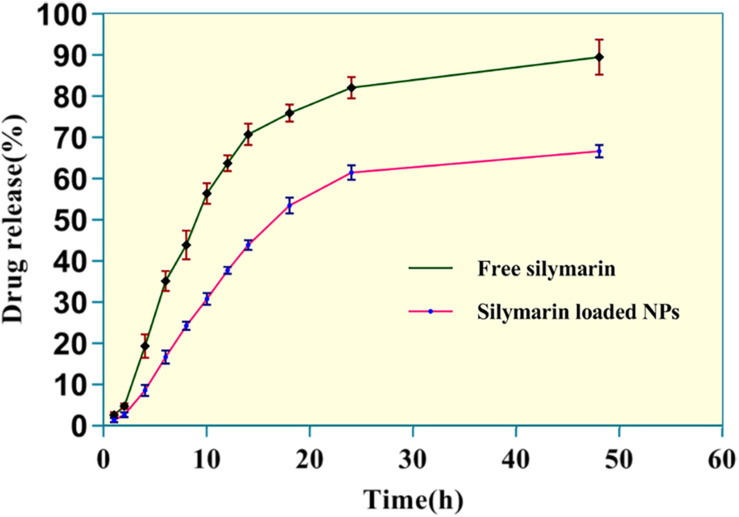
The *in vitro* release kinetics profile of free silymarin and silymarin encapsulated in collagen nanoparticle. The experiment was conducted for *n* = 5 data sets.

*In vitro* release patterns of free silymarin and silymarin encapsulated in collagen nanoparticles are shown in Figure 4. The *in vitro* release profile was observed for 48 h for both free silymarin and silymarin encapsulated in collagen nanoparticles. In the first 10 h, about 30.72 ± 1.40% of the drug from collagen nanoparticles was released in comparison to 56.32 ± 2.56% of free drug released. In the first 18 h, about 53.32 ± 1.90% of the drug was released from nanoparticle in comparison to 75.76 ± 2.12% of the free drug, thus showing a slow and sustained release profile over 48 h of study. The release kinetics showed a slow and controlled release of silymarin from collagen nanoparticles into the phosphate buffer at a physiological pH 7.4 in comparison to the release of free silymarin under similar conditions (Figure 4).

#### Cell Viability Assay

The cytotoxicity profile of blank NPs, silymarin-loaded NPs, and silymarin was determined using MTT assay on HUVECs at varying concentrations (0.025, 0.25, 2.5, 25, 50, 100, and 200 μg/ml). The percentage of cell viability was measured with respect to untreated control cells. From the results of MTT assay, it was observed that at lower concentrations <25 μg/ml, cells treated with blank NPs, silymarin-loaded NPs, and silymarin showed more than 94% cell viability. At a concentration of 25 μg/ml, cell viability of 76.27 ± 3.04% was observed to silymarin-exposed cells. A significant increase in the cytotoxicity was observed at higher concentrations of silymarin with 39.42 ± 3.15% cell viability observed at 200 μg/ml concentration of silymarin. For concentrations >100 μg/ml, no significant change in cytotoxic effect for silymarin-exposed cells was observed. A lesser cytotoxic effect was observed with silymarin-loaded NPs compared to silymarin-treated group with 58.27 ± 3.03% cell viability observed at 50 μg/ml concentration of silymarin. The order of increase in cytotoxicity was blank NPs < silymarin-loaded NPs < silymarin for all the concentrations. The figure for MTT assay has been included in the Supplementary Figure 1.

### *In vivo* Studies Results

#### Neurobehavioral Parameters Analysis

##### Rota-rod

**FIGURE 5:**
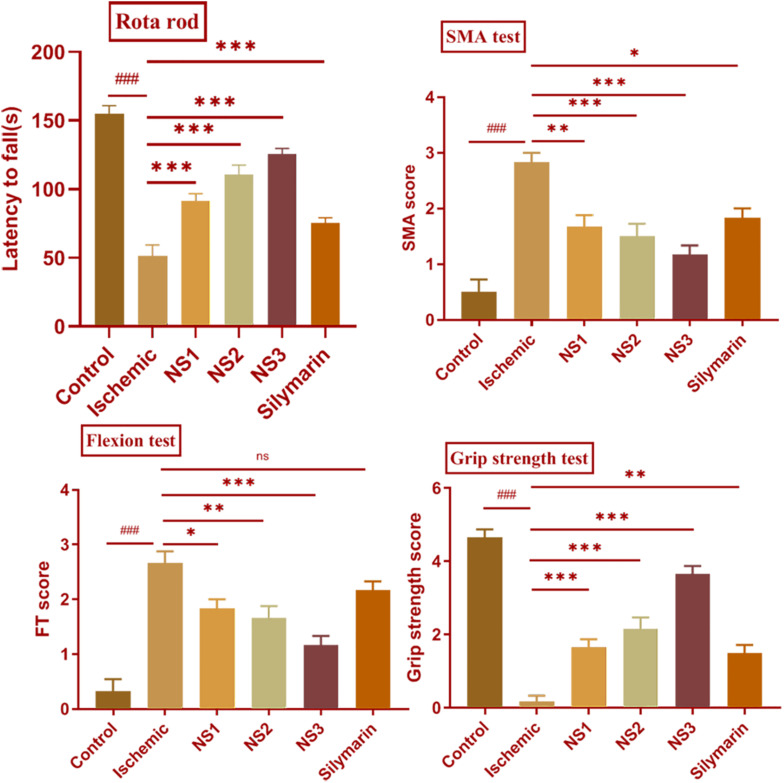
Effect of nanosilymarin on neurobehavioral deficits like rota-rod, spontaneous motor activity (SMA), flexion test (FT), and grip strength test for control (C), ischemic (I), NS1 (10 μg/kg b.wt), NS2 (100 μg/kg b.wt), NS3 (1,000 μg/kg b.wt), and free silymarin (Sil) (100 mg/kg b.wt). The neurological deficit was significant in the ischemic (I) group in comparison to the control (C) group. Treating the animals with nanosilymarin has decreased neurological deficit, especially at NS3 dose. Values are expressed as mean ± SEM. Results on comparison with the control group showed a significant difference (^###^*p* < 0.001). Results obtained showed significant difference from ischemic group (^ns^*p* > 0.05, ^∗^*p* < 0.05, ^∗∗^*p* < 0.01, and ^∗∗∗^*p* < 0.001). The experiment was conducted for *n* = 6 data sets and results represented as mean ± SEM.

In the MCAO group, there was an evident motor impairment function due to cerebral ischemia. The diseased (MCAO) group showed significantly reduced muscular coordination (51.17 ± 3.39, *p* < 0.001) skill in comparison to the control group. Animals pretreated with NS1 + MCAO (91.33 ± 2.22, *p* < 0.001), NS2 + MCAO (110.70 ± 2.81, *p* < 0.001) and NS3 + MCAO (125.50 ± 1.73, *p* < 0.001) showed a significantly improved muscular coordination skill in comparison to MCAO group animals with the NS3-treated group showing best results. The free silymarin-treated group showed a significantly improved muscular coordination (75.17 ± 1.66, *p* < 0.001) in comparison to the MCAO group. All three nanosilymarin-treated groups showed improvement in muscular coordination as compared to the free silymarin group (Figure 5; Supplementary Table 2).

##### SMA test analysis

Spontaneous motor activity analysis showed a lack of neurological deficits in control group rats, whereas diseased (MCAO) group rats showed severe neurological deficits. MCAO group rats did not show any movement and had their posture bent toward the paretic side. There was a significant loss in the SMA efficiency (2.83 ± 0.17, *p* < 0.001) in MCAO group rats in comparison to the control group rats. The treatment groups NS1 + MCAO (1.67 ± 0.21, *p* < 0.01), NS2 + MCAO (1.50 ± 0.22, *p* < 0.001), and NS3 + MCAO (1.17 ± 0.17, *p* < 0.001) showed significant improvement in SMA score in comparison to the MCAO group rats with the NS3 treatment group showing highly improved neurological scores in comparison to other nanosilymarin-treated groups. There was a significant difference in the SMA score (1.83 ± 0.17, *p* < 0.05) of the free silymarin group in comparison to the MCAO group. SMA score of free silymarin-treated group was low in comparison to all three nanosilymarin-treated groups (Figure 5; Supplementary Table 3).

##### Flexion test

In comparison to the control group, the flexion test showed a significantly high score in the MCAO rats (2.67 ± 0.21, *p* < 0.001). Neurological deficit was significantly decreased in NS1 + MCAO (1.83 ± 0.17, *p* < 0.05), NS2 + MCAO (1.67 ± 0.21, *p* < 0.01), and NS3 + MCAO (1.17 ± 0.17, *p* < 0.001) treated groups when compared with the MCAO group rats. In the treatment group, NS3 + MCAO neurological deficit was restored near to the control group rats. The difference in neurological score between free silymarin-treated group and MCAO group animals was non-significant (2.17 ± 0.17, *p* > 0.05), indicating not much reduction in reducing the neurological deficit (Figure 5; Supplementary Table 4).

##### Grip strength test

There was a significantly decreased grip strength (0.17 ± 0.17, *p* < 0.001) in the MCAO group animals in comparison to the control group rats. The grip strength score of all the three nanosilymarin-treated groups NS1 + MCAO (1.67 ± 0.21, *p* < 0.001), NS2 + MCAO (2.17 ± 0.31, *p* < 0.001), and NS3 + MCAO (3.67 ± 0.21, *p* < 0.001) showed a significant rise in comparison to the MCAO group rats showing a considerable improvement in restoring grip strength near to the control group rats. The free silymarin-treated group showed a significant grip strength score (1.50 ± 0.22, *p* < 0.01) in comparison to the MCAO group rats. The grip strength showed remarkable improvement in the nanosilymarin-treated groups in comparison to free silymarin with the NS3-treated group showing highly improved grip strength (Figure 5; Supplementary Table 5).

#### Evaluation of Infarct Size (%)

**FIGURE 6:**
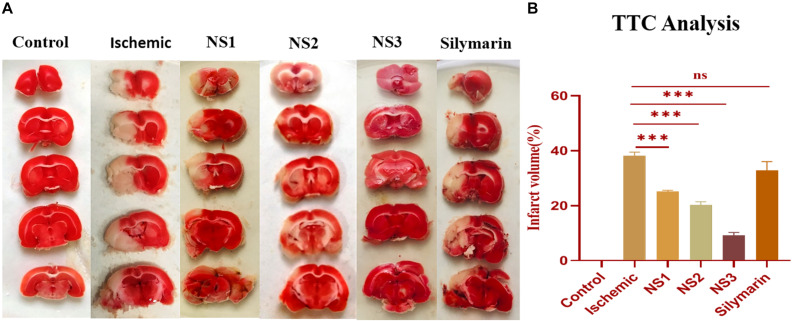
**(A)** Photographs showing effect of nanosilymarin on brain infarct in control (C), ischemic (I), NS1 (10 μg/kg b.wt), NS2 (100 μg/kg b.wt), NS3 (1,000 μg/kg b.wt), and free silymarin (Sil) (100 mg/kg b.wt), respectively. Infarct region is very well visible in the ischemic group (I) brain sections as compared to the control (C) group brain sections, and all the three treatment groups showed a significant reduction in infarct region in comparison to free silymarin group. **(B)** Quantification of infarct analysis. The experiment was conducted for *n* = 3 data sets, and results are shown as mean ± SEM. Results on comparison with the control group showed a significant difference (^###^*p* < 0.001). Results showed a significant difference from the ischemic group (^ns^*p* > 0.05 and ^∗∗∗^*p* < 0.001).

TTC staining of brain sections was done to assess the extent of infarction in different groups. The infarct volume showed a significant variation among all the groups. The TTC staining of ipsilateral sections of the MCAO group rats showed a significantly higher infarct volume in comparison to the control group rats (38.08 ± 1.39%, *p* < 0.001) (Figures 6A,B). TTC analysis showed a significant reduction in infarct volume in the NS1 + MCAO group (25.08 ± 0.47%, *p* < 0.001), NS2 + MCAO group (20.23 ± 1.19%, *p* < 0.001), and NS3 + MCAO group (9.23 ± 1.03%, *p* < 0.001) in comparison to the MCAO group. The reduction in infarct volume in Sil + MCAO group (32.95 ± 1.78%, *p* > 0.05) was non-significant in comparison to the MCAO group. The results demonstrated that reduction in infarction was more in nanosilymarin-treated groups in comparison to the silymarin-treated group, with the NS3 + MCAO group showing the best results of all the nanosilymarin treatments.

#### Biochemical Parameter Analysis

##### TBARS assay results

**FIGURE 7:**
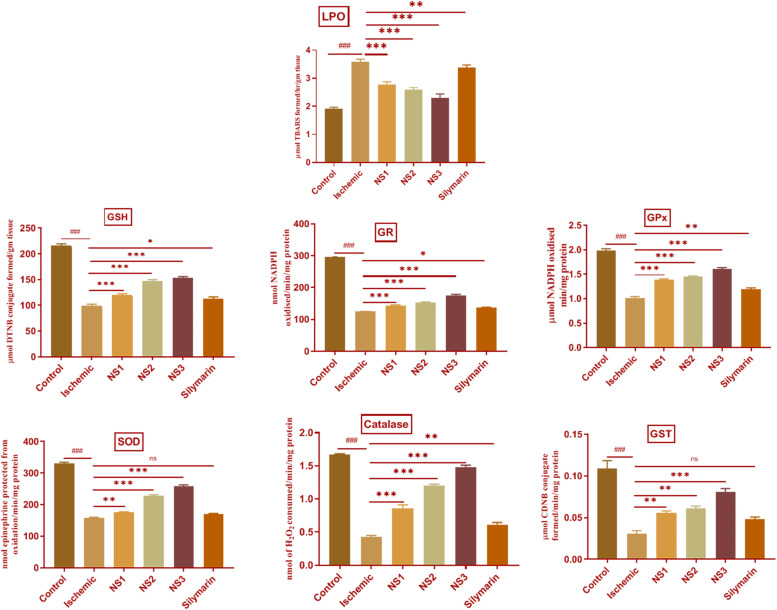
Effect of nanosilymarin formulation on various biochemical markers like lipid peroxidation (LPO), glutathione (GSH), glutathione reductase (GR), glutathione peroxidase (GP_X_), superoxide dismutase (SOD), glutathione-S-transferase (GST), and catalase (CAT). Groups shown are control (C), ischemic (I), NS1 (10 μg/kg b.wt), NS2 (100 μg/kg b.wt), NS3 (1,000 μg/kg b.wt), and silymarin (Sil) (100 mg/kg b.wt). A significant increase in LPO content and a decrease in other antioxidant marker content can be seen in the ischemic (I) group in comparison to the control (C) group. Treatment with NS3 restored the marker content significantly. The experiment was conducted for *n* = 6 data sets, and results are shown as mean ± SEM. Results on comparison with the control group showed a significant difference (^###^*p* < 0.001). Results obtained showed significant difference from the ischemic group (^∗^*p* < 0.05, ^∗∗^*p* < 0.01, ^∗∗∗^*p* < 0.001, and ^ns^*p* > 0.05).

The extent of oxidative damage was measured by estimation of TBARS content. The extent of lipid peroxidation can be detected by TBARS assay by using thiobarbituric acid. It was observed that there was a significant (3.59 ± 0.04, *p* < 0.001) increase in the level of TBARS content in the MCAO group rats (Figure 7; Supplementary Table 6) in comparison to the control group rats. The rats of NS1 + MCAO (2.78 ± 0.04, *p* < 0.001), NS2 + MCAO (2.59 ± 0.03, *p* < 0.001), and NS3 + MCAO (2.31 ± 0.05, *p* < 0.001) groups showed significantly reduced TBARS content in comparison to the MCAO group. The free silymarin-treated group showed a significant difference (3.37 ± 0.04, *p* < 0.01) in TBARS content in comparison to the MCAO group. Nanosilymarin-treated groups showed more reduced TBARS content than the free silymarin-treated group. Treatment group NS3 + MCAO showed maximum attenuation to the level of TBARS and restored it near to the control group.

##### Estimation of GSH content

The level of GSH content showed a significant (98.99 ± 2.92, *p* < 0.001) depletion in the MCAO group rats in comparison to the control group, as shown in Figure 7 (Supplementary Table 11). Nanosilymarin treatments of NS1 + MCAO (119.7 ± 2.70, *p* < 0.001), NS2 + MCAO (147.50 ± 2.29, *p* < 0.001), and NS3 + MCAO (152.80 ± 2.88, *p* < 0.001) showed significantly increased GSH levels in comparison to the MCAO group. The free silymarin-treated group showed significantly (112.90 ± 3.23, *p* < 0.05) elevated levels of GSH in comparison to the MCAO group. The increase in GSH content in the nanosilymarin-treated groups was more than that in the free silymarin-treated group. A maximum increase in GSH content was observed in the NS3 + MCAO group among all three nanosilymarin-treated groups.

##### Activities of antioxidant enzymes

The antioxidant enzymes GR (124.40 ± 0.78, *p* < 0.001), GP_X_ (1.02 ± 0.03, *p* < 0.001), SOD (159.0 ± 1.5, *p* < 0.001), catalase (0.43 ± 0.02, *p* < 0.001), and GST (0.030 ± 0.004, *p* < 0.001) showed significantly decreased activities in the MCAO group rats in comparison to the control group rats as shown in Figure 7 (Supplementary Tables 7–10, 12). The activities of all antioxidant enzymes showed a significant GR (143.0 ± 2.8, *p* < 0.001 | 152.30 ± 2.2, *p* < 0.001 | 174.1 ± 4.8, *p* < 0.001), GP_X_ (1.38 ± 0.02, *p* < 0.001 | 1.45 ± 0.02, *p* < 0.001 | 1.61 ± 0.03, *p* < 0.001), SOD (175.3 ± 2.1, *p* < 0.01 | 228.8 ± 2.8, *p* < 0.001 | 259.0 ± 3.7, *p* < 0.001), catalase (0.86 ± 0.05, *p* < 0.001 | 1.20 ± 0.02, *p* < 0.001 | 1.48 ± 0.03, *p* < 0.001), and GST (0.056 ± 0.002, *p* < 0.01 | 0.061 ± 0.002, *p* < 0.01| 0.080 ± 0.004, *p* < 0.001) increase in all the three nanosilymarin-treated groups NS1 + MCAO, NS2 + MCAO, and NS3 + MCAO in comparison to the MCAO group rats. The free silymarin-treated group showed improved GR (136.5 ± 2.1, *p* < 0.05), GP_X_ (1.19 ± 0. 03, *p* < 0.01), SOD (170.3 ± 2.2, *p* > 0.05), catalase (0.61 ± 0.03, *p* < 0.01), and GST (0.048 ± 0.003, *p* > 0.05) activities of all antoxidant enzymes in comparison to the MCAO group. All the nanosilymarin-treated groups showed increased activities of all antioxidant enzymes in comparison to the free silymarin group. Maximum increase in antioxidant enzymes activity was observed in the NS3-treated group in comparison to other nanosilymarin-treated groups.

#### Analysis of Histopathological Changes

**FIGURE 8:**
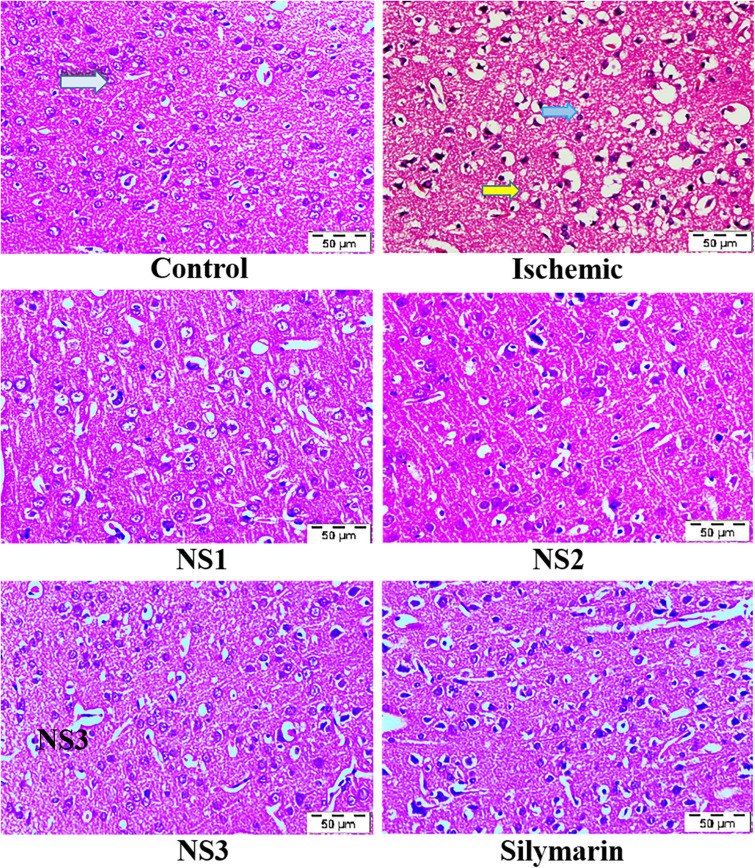
Photographs showing histopathological changes (40×) in the cortex region for all the groups, i.e., control (C), ischemic (I), NS1 (10 μg/kg b.wt), NS2 (100 μg/kg b.wt), NS3 (1,000 μg/kg b.wt), and free silymarin (Sil) (100 mg/kg b.wt). The control (C) group showed intact neurons (light blue arrow) and normal morphology. In contrast, in the ischemic (I) group, degenerative changes like neuronal loss (yellow arrow), vacuolation (loss of intact neurons shown by the dark blue arrow), and altered morphology are visible. The NS1 treatment group showed decreased vacuolation and fewer morphological changes. These results are further improved in NS2 and NS3 when compared with the ischemic (I) and free silymarin treatment group.

The histopathological changes in hematoxylin and eosin-stained coronal sections of the brain observed after induction of MCAO and 24 h reperfusion are shown in Figure 8. The frontal cortex region of brain sections was examined for histopathological changes. In the histopathology of the control group brain sections, normal neurons with no visible pathological changes can be observed. Brain sections of the MCAO group rats showed neuronal loss with vacuolated spaces in between. Brain sections of groups treated with nanosilymarin doses of NS1 + MCAO, NS2 + MCAO, and NS3 + MCAO exhibited a partial neuronal loss along with the presence of intact neurons in between vacuolated spaces. This showed restoration of histopathology near to the control group.

#### Analysis of Immunohistopathological Changes

**FIGURE 9:**
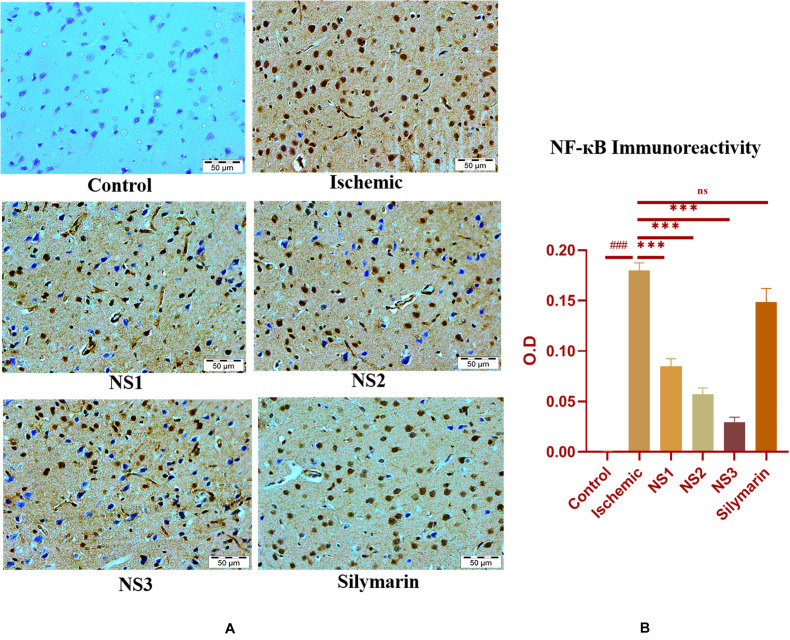
**(A)** Photograph (40×) shows immunohistochemical expression of nuclear factor kappa B (NF-κB) in middle cerebral artery occlusion (MCAO) model in the control (C), ischemic (I), and treatment groups of nanosilymarin formulations NS1 (10 μg/kg b.wt), NS2 (100 μg/kg b.wt), NS3 (1,000 μg/kg b.wt), and free silymarin (Sil) (100 mg/kg b.wt). The control (C) group showed no expression for the NF-κB marker, whereas the ischemic (I) group shows maximum expression. Treatments NS1 and NS2 showed moderate expression but less than free silymarin; NS3 shows minimum expression. **(B)** Quantification of NF-κB expression intensity as optical density (OD). The experiment was conducted for *n* = 3 data sets, and results are shown as mean ± SEM. Results on comparison with the control group showed a significant difference (^###^*p* < 0.001). Results obtained showed significant difference from the ischemic group (^∗∗∗^*p* < 0.001 and ^ns^*p* > 0.05).

**FIGURE 10:**
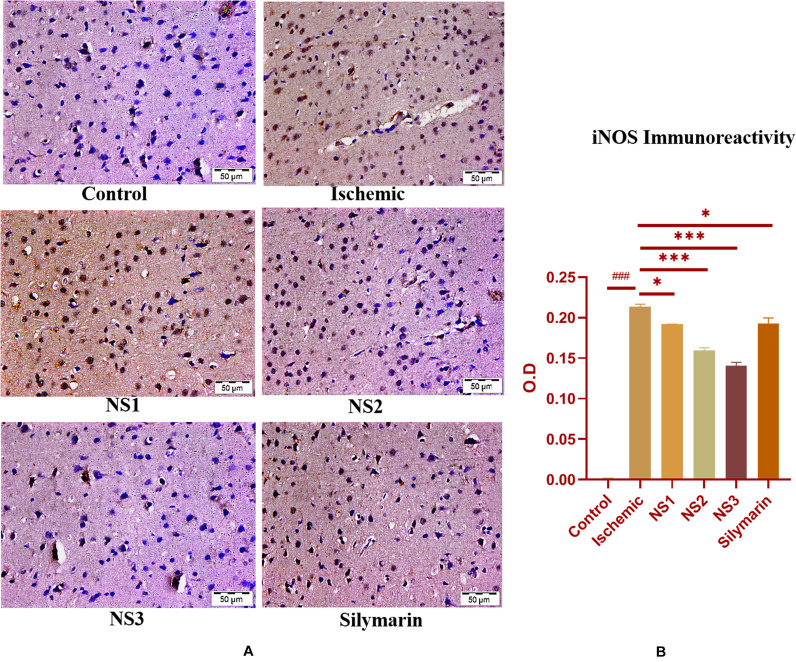
**(A)** Photograph (40×) shows immunohistochemical expression of inducible nitric oxide synthase (iNOS) in middle cerebral artery occlusion (MCAO) model in control (C), ischemic (I), and treatment groups of nanosilymarin formulations NS1 (10 μg/kg b.wt), NS2 (100 μg/kg b.wt), NS3 (1,000 μg/kg b.wt), and free silymarin (Sil) (100 mg/kg b.wt). The control (C) group showed almost no expression for the iNOS marker, whereas the ischemic (I) group shows maximum expression. Treatments NS1 and NS2 showed moderate to mild expression but less than free silymarin; NS3 shows minimum expression. **(B)** Quantification of iNOS expression intensity as optical density (OD). The experiment was conducted for *n* = 3 data sets, and results are shown as mean ± SEM. Results on comparison with the control group showed a significant difference (^###^*p* < 0.001). Results obtained showed significant difference from the ischemic group (^∗∗∗^*p* < 0.001 and ^∗^*p* < 0.05).

**FIGURE 11:**
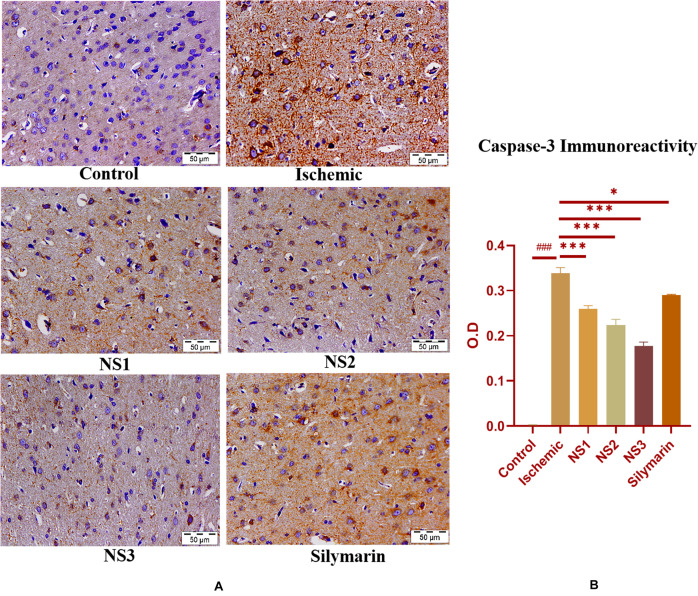
**(A)** Photograph (40×) shows immunohistochemical expression of caspase-3 in the middle cerebral artery occlusion (MCAO) model in control (C), ischemic (I), and treatment groups of nanosilymarin doses NS1 (10 μg/kg b.wt), NS2 (100 μg/kg b.wt), NS3 (1,000 μg/kg b.wt), and free silymarin (Sil) (100 mg/kg b.wt). The control (C) group shows almost no expression for the caspase-3 marker, whereas the ischemic (I) group shows maximum expression. Treatments NS1 and NS2 showed moderate to mild expression but less than free silymarin; NS3 showed minimum expression. **(B)** Quantification of caspase-3 expression intensity as optical density (OD). The experiment was conducted for *n* = 3 data sets, and results are shown as mean ± SEM. Results on comparison with the control group showed a significant difference (^###^*p* < 0.001). Results obtained showed significant difference from the ischemic group (^∗∗∗^*p* < 0.001 and ^∗^*p* < 0.05).

The expression of inflammatory proteins NF-κB, iNOS, and apoptotic protein caspase-3 was examined in hematoxylin and DAB-stained brain sections. The protein expression has been presented in the form of a histogram in which the intensity of DAB staining for each group was plotted as optical density (OD). Quantification data showed significantly increased expression of inflammatory marker NF-κB (more OD) in the MCAO group in comparison to the control group (0.180 ± 0.007, *p* < 0.001) (Figures 9A,B; Supplementary Table 13). The control group showed almost negligible expression of NF-κB. The expression of NF-κB in nanosilymarin-treated groups NS1 + MCAO (0.085 ± 0.007, *p* < 0.001), NS2 + MCAO (0.057 ± 0.006, *p* < 0.001), and NS3 + MCAO (0.029 ± 0.005, *p* < 0.001) showed significant reduction in expression (less OD) in comparison to the MCAO group. Whereas free silymarin-treated group Sil + MCAO did not show any significant difference in expression (0.149 ± 0.013, *p* > 0.05) when compared to the MCAO group. The nanosilymarin-pretreated group NS3 showed reduced expression near to the control group, followed by NS2 and NS1. However, the free silymarin-treated group did not show much reduction in expression in comparison to the nanosilymarin-treated groups. The inflammatory marker iNOS showed a remarkably increased expression (0.213 ± 0.003, *p* < 0.001) in the MCAO group in comparison to the control group (Figures 10A,B; Supplementary Table 14). The control group showed nil or almost negligible expression. The expression of iNOS in nanosilymarin-treated groups NS1 + MCAO (0.192 ± 0.000, *p* < 0.05), NS2 + MCAO (0.160 ± 0.003, *p* < 0.001), and NS3 + MCAO (0.140 ± 0.004, *p* < 0.001) showed significant reduction in expression in comparison to the MCAO group. The free silymarin-treated group Sil + MCAO showed a significant difference in expression (0.193 ± 0.006, *p* < 0.05)

when compared to the MCAO group. The nanosilymarin-pretreated group NS3 showed reduced expression near to the control group, followed by NS2 and NS1. However, there was no significant difference in the expression of iNOS between free silymarin-treated group and NS1 group. However, a significant reduction in the iNOS expression was observed in the nanosilymarin-treated groups in comparison to free silymarin-treated group. The expression of apoptotic marker caspase-3 was significantly elevated (0.338 ± 0.012, *p* < 0.001) in the MCAO group in comparison to the control group (Figures 11A,B; Supplementary Table 15). The control group showed nil or almost negligible expression. The expression of caspase-3 in nanosilymarin-treated groups NS1 + MCAO (0.259 ± 0.008, *p* < 0.001), NS2 + MCAO (0.224 ± 0.012, *p* < 0.001) and NS3 + MCAO (0.177 ± 0.009, *p* < 0.001) showed significant reduction in expression in comparison to the MCAO group. The free silymarin-treated group Sil + MCAO showed a significant difference in expression (0.290 ± 0.001, *p* < 0.05) when compared to the MCAO group. The nanosilymarin-pretreated group NS3 showed reduced expression near to the control group, followed by NS2 and NS1. However, the free silymarin-treated group did not show much reduction in expression in comparison to the nanosilymarin-treated groups.

Brain sections of the MCAO group rats exhibited increased activity of apoptotic marker caspase-3, as shown in Figure 11. Treatment with nanosilymarin attenuated its expression, with the NS3 + MCAO-treated group showing minimum expression and Si + MCAO-treated group showing high expression but less than the MCAO group.

## Discussion

In the current investigation, we aimed to search for a better therapeutic approach by comparing the effectiveness of free silymarin and nanosilymarin in countering the oxidative stress and inflammation induced by I/R injury in rat brains. Most of the previous studies involving the use of silymarin for treating I/R-based injury required administering an unusually high dose of silymarin due to its poor bioavailability (Ghosh et al., 2010; Javed et al., 2011). However, the current study has very well demonstrated an enhancement in the therapeutic efficacy of silymarin at 100, 1,000, and 10,000 times lower concentrations when administered as a nanoformulation. There was a significant improvement in neuroprotective effect, as was evident by improved neurological functioning and motor performance, decreased cerebral infarction, and desirable alterations in related biochemical parameters.

It has been widely reported that the size and shape of formulated nanoparticle plays a crucial role in a drug loading, release, targeting, and particle absorption (Gupta et al., 2014; Chenthamara et al., 2019). Nanoparticle-aided drug delivery has been associated with the biggest constraint involving clearance by the RES through opsonization. This makes the role of size even more important, as it influences clearance as well as the distribution of the particle. If the particle size exceeds 100 nm, its biodistribution and pharmacokinetic properties change, and it can be easily detected in the bloodstream and organs like the liver, lungs, kidney, and spleen (De Jong et al., 2008). The characterization results for silymarin-encapsulated collagen nanoparticle obtained from TEM image and DLS analysis confirmed the size of the particle as 44.80 ± 3.83 nm with a PDI of 0.299 ± 0.024 having a spherical morphology. FT-IR analysis confirmed the synthesis of collagen nanoparticles. The size of the prepared nanoparticle was optimum to avoid clearance by RES, thus increasing its blood circulation time. Prepared collagen nanoparticle had an entrapment efficiency of 76.7 ± 2.4% and a loading efficiency of 3.17 ± 0.37%, and it exhibited a slow and sustained drug release in drug release kinetics study. Drug release by a drug delivery system depends on the loading and subsequent release at the site of action. Particle size influences drug release, as small particles have a higher surface area to volume ratio. Therefore, most of the drug associated with nanoparticle stays at the surface, leading to faster drug release. In contrast, larger particles have large cores allowing more drug encapsulation and slower release (Redhead et al., 2001), while slow and sustained drug release from the collagen nanoparticle may be due to the crosslinking of collagen nanoparticle leading to the formation of the collagen matrix. The drug gets entrapped in the interstices of the collagen matrix, thus showing slow and sustained release (Kukkar et al., 2017).

3-(4,5-dimethylthiazol-2-yl)-2, 5-diphenyl tetrazolium bromide assay was performed to evaluate the *in vitro* toxicity of silymarin-loaded NPs in comparison with blank NPs and silymarin on HUVECs. Exposure of HUVEC cells to different concentrations of silymarin showed dose-dependent cytotoxicity. Silymarin exhibited the least cytotoxicity at lower concentrations. However, a significant reduction in cell viability was observed above a concentration of 2.5 μg/ml. The results were in accordance with the previous report (Vakili Zahir et al., 2018). Blank NPs were observed to be least cytotoxic even at the highest concentrations tested, which can be attributed to the biocompatible and biodegradable nature of the excipients used in the formulation development (Chan et al., 2020). Silymarin-loaded NPs showed less cytotoxicity in comparison to free silymarin, as the drug has been efficiently entrapped inside the collagen nanoparticle and is slowly released from the nanoparticle resulting in less drug exposure to cells under similar conditions. It was evident from the cytotoxicity results that the developed nanoparticles were compatible with the normal cells and hence safe for use as drug carriers.

Focal occlusion of MCA has been reported to be the most common cause of stroke in humans. Animal models of focal cerebral ischemia are the most relevant when it comes to mimicking human stroke pathophysiology (Sommer, 2017). Evaluation of various behavioral parameters showed considerable neurological dysfunction and motor impairment in the MCAO group. The motor dysfunction arises out of damage inflicted upon the motor cortex and pyramidal tracts, which are present in the middle cerebral artery region. This leads to a blockage of electrical activity in the subcortical regions and irrecoverable damage to synaptic activity in the motor cortex region (Hua et al., 2019). Occluding MCA in rats induces infarction in the territory being supplied by MCA, including the cerebral cortex of frontal, sensorimotor, and segments of caudate-putamen, which regulates the motor and sensorimotor activities. This results in various sensorimotor and motor deficits like reduced locomotion, lack of coordination, and partial paralysis (Schaar et al., 2010). Furthermore, the induction of cerebral ischemia generates free radicals that play a key role in neurobehavioral deficits through oxidative stress (Fukui et al., 2002). Thus, the oxidative stress induced by free radical generation might be responsible for the poor neurobehavioral outcome of the MCAO group rats. In the current investigation, we observed that motor and sensorimotor activities, muscular coordination skills, and grip strength were severely impaired in the MCAO group rats. This might be due to the generation of free radicals leading to oxidative stress in the frontal cortex and striatum, which controls the motor and sensory activities (Raza et al., 2011a). Furthermore, in the current study, rats were assessed for several neurobehavioral parameters like rota-rod, SMA, flexion test, and grip strength for determining the neurological deficit and motor coordination after induction of focal cerebral ischemia. Several earlier studies reported that pretreatment with silymarin showed improvement in various behavioral outputs (Raza et al., 2011b). Pretreatment with nanosilymarin showed significant restoration of neurological deficits and motor coordination in comparison to the MCAO group. The results obtained for nanosilymarin treatments NS1, NS2, and NS3 showed a considerable improvement in comparison to the free silymarin-treated group. Thus, the outcomes of behavioral studies confirmed that silymarin encapsulation into collagen nanoparticle enhanced its efficacy by increasing bioavailability.

It has already been reported that after MCAO, the extent of injury to the cortex, striatum, and hippocampus regions depends upon the time of MCA occlusion and reperfusion period (Heurteaux et al., 2006; Rathore et al., 2020). The brain infarct volume was measured after I/R injury by staining the coronal brain sections using TTC, a compound that is reduced into pink formazan by mitochondrial succinate dehydrogenase activity in non-ischemic tissue. Under ischemic–hypoxia conditions, the energy demand of cells rises, putting mitochondria under high stress. This decreases the activity of succinate dehydrogenase enzyme; thus, it functions as a stress marker for ischemic–hypoxia conditions. MCAO group rat brains showed prominent infarct size due to oxidative stress induced by neuronal loss. Pretreatment with silymarin has been reported to show the neuroprotective effect by reducing brain infarct. This may be accredited to a decrease in neuronal damage and an improved antioxidant defense system (Nencini et al., 2007). In the current study, the MCAO group showed prominent and visible infarct constricted to the cortex and striatum region along with some areas of the hippocampus. There was a significant reduction in infarct size on pretreatment with nanosilymarin doses NS1, NS2, and NS3 in comparison to the MCAO group. In addition, in the nanosilymarin-treated groups, decrease in infarct was more when compared to free silymarin-treated groups. This showed that encapsulating silymarin into collagen nanoparticles enhanced its neuroprotective effect in comparison to free silymarin, as much better therapeutic effects were obtained at much lower doses. I/R-based brain injury results in biochemical alterations due to oxidative stress. A burst of free radicals generated plays an important role in neuronal damage due to their high reactivity and capacity to cause cellular damages (Aprioku, 2013). Reactive oxygen species attack lipid-rich membranes to cause lipid peroxidation (Ayala et al., 2014). In the current investigation, elevated levels of LPO in the form of TBARS and reduced levels of GSH content were observed. The excessive accumulation of glutamate in the extracellular fluid causes hyperexcitability of neurons in the initial phase of the postischemic period; this can induce activation of N-methyl-D-aspartate (NMDA) in excess, resulting in the accumulation of sodium and calcium ions and intracellular fluids. This results in the generation of free radicals and lipid peroxides. An antioxidant system, GSH, is present in all the animal cells; it protects against singlet oxygen, superoxide, and hydroxyl-radical-induced damage by reacting with free radicals (Sinha et al., 2001). Overproduction of free radicals after ischemic brain injury causes oxidative damage to the level of lipids and proteins in the membrane. This results in reduced GSH content and enzymes like GR, GP_X_, and SOD dependent on it. Superoxide and its derivatives destroys cells in a variety of ways, after the ischemic injury. Superoxide dismutase contains the maximum burden of free radicals in ischemic reperfusion insult. SOD plays a crucial role in negating the effect of oxidative stress, as it catalyzes the dismutation of superoxide anions to hydrogen peroxides. The combined antioxidant enzyme system containing SOD, GR, GP_X_, and catalase together are involved in defense mechanisms when cells are exposed to oxygen (Zhan and Yang, 2006). Catalase converts hydrogen peroxide and oxygen to water. Previous findings have found that pretreatment with silymarin has resulted in countering the alterations in markers of oxidative stress (Baluchnejadmojarad et al., 2010; Chtourou et al., 2010). Pretreatment with nanosilymarin showed significantly improved results in comparison to free silymarin at much lower concentrations, as was evident by findings of the study. It is thus confirming the improved efficacy of silymarin by nanoencapsulation in collagen nanoparticles.

Middle cerebral artery occlusion results in oxidative stress due to free radical generation that further leads to depletion of GSH content, elevation in LPO, and ionic imbalance (Raza et al., 2011b). All these factors contribute to the neuronal loss, as observed in the histopathological study of the brain. Histopathological changes like neuronal loss, swelling of neurons, and vacuolated spaces were found in the cortical region following MCAO in rats. Pretreatment with silymarin showed partial improvement in the histopathological alterations, thus conforming neuroprotection by silymarin (Muley et al., 2012). These changes were restored near to the normal histoarchitecture on pretreatment with nanosilymarin in comparison to the free silymarin-treated group. Cerebral ischemia induction by MCAO activates certain inflammatory molecules like NF-κB and iNOS that eventually leads to cell death (Ji et al., 2011). Immunohistochemistry studies were performed to evaluate the extent of inflammation after I/R injury following an ischemic stroke during the early hours. NF-κB, a transcription factor, not only plays a vital role in the pathophysiology of ischemic stroke but also in cell survival and inflammation. It can be activated in neurons by hypoxia, inflammatory mediators, and ROS (Ridder and Schwaninger, 2009). The anti-inflammatory effect of silymarin is due to one of its active constituent, silybin, which downregulates the expression of NF-κB by preventing its activation (Wang et al., 2012), as NF-κB is also a key regulator of the inducible expression of iNOS. Prevention of NF-κB activation further downregulates the expression of iNOS, thus adding to the cytoprotection. This modulation of NF-κB by silymarin results in the reduction in oxidative as well as nitrosative stress by preventing the generation of ROS and NO, thus preventing neuronal death (Borah et al., 2013). Upregulation of iNOS as inflammatory response results in excessive production of NO and prostaglandins, which further aggravates the damage (Krakauer, 2004; Yokota et al., 2004; Vaibhav et al., 2012). Increased production of NO disrupts cellular microvascular integrity and causes edema formation (Vaibhav et al., 2012). Silymarin is also known to downregulate the expression of iNOS (Kahle et al., 2009). In accordance with earlier findings, the present study showed overexpression of inflammatory markers NF-κB and iNOS in the MCAO group rats. Pretreatment with nanosilymarin significantly decreased the expression of NF-κB and iNOS, thus attenuating inflammatory damage in comparison to the MCAO group. The free silymarin-treated group showed reduced expression of NF-κB and iNOS, but the decrease was more prominent in the nanosilymarin-treated group. The characteristic outcome of this study was that a significant anti-inflammatory effect of silymarin encapsulated collagen nanoparticle was found when compared to free silymarin. It has been well established that ischemic reperfusion injury causes cerebral injury by inducing neuronal loss due to apoptosis (Broughton et al., 2009; Wang et al., 2009). After focal cerebral ischemia induction, cells present in the core region of brain tissue undergoes necrosis due to sudden exposure to reduced blood flow. The penumbra region surrounding the necrotic core is highly susceptible to apoptosis. The induction of focal cerebral ischemia by MCAO involves DNA damage, which results in the induction of p53-controlled release of cytochrome c from the mitochondria, which further binds to apoptosis activating factor-1 (Apaf-1) to form apoptosomes. This leads to the activation of caspase-9, which further activates effector caspase-3, thus resulting in apoptosis-mediated cell death. Therefore, the pathophysiology of stroke-induced apoptotic neuronal cell death involves induction of p53, Apaf-1, and caspase-3 activation and ischemic injury is reduced by inhibition of p53, Apaf-1 and caspases expression (Fortin et al., 2001; Khan et al., 2010). It has already been reported that silymarin impedes the activation of the apoptotic pathway by preventing apoptosome formation by inhibiting Apaf-1 that controls the activation of caspases (Raza et al., 2011b; Borah et al., 2013). The inhibition of activation of p53, Apaf-1, and caspases due to antiapoptotic properties of silymarin reduces ischemic stroke brain injury. The results of this study showed an increased expression of apoptotic marker caspase-3 in the MCAO group. Pretreatment with nanosilymarin resulted in a significant reduction in caspase-3 expression in comparison to the MCAO group. The suppression in caspase-3 expression was more in the nanosilymarin-treated group when compared to the free silymarin group. The outcome of this study showed that the encapsulation of silymarin into collagen nanoparticles had a marked improvement in its antiapoptotic property.

To summarize, the findings of this study demonstrate an enhanced neuroprotective effect of silymarin after nanoencapsulation in collagen nanoparticles. The experimental data show that nanoencapsulation of silymarin significantly ameliorates ischemia/reperfusion-induced rat brain injury by reducing neurological deficits and oxidative damage followed by suppression of inflammatory and apoptotic responses. These observations indicate that nanosilymarin may be a viable protective agent for the treatment of various CNS diseases where cellular dysfunction is a result of oxidative stress.

## Conclusion

The outcomes of the present study have suggested that the already established neuroprotective effect of silymarin due to its anti-inflammatory, antioxidant, and antiapoptotic properties can be well harnessed to ameliorate ischemic brain injury. Pretreatment with silymarin encapsulated into a collagen-based nanoparticle drug delivery system enhanced the therapeutic efficacy of silymarin against ischemia/reperfusion injury by increasing its bioavailability. The nanoparticle surface can be further multifunctionalized to achieve the desired property and can pave the way for a new field of research in treating various neuroprotective diseases. In the present investigation, we have evaluated the neuroprotective effect of silymarin-encapsulated collagen nanoparticles for an acute ischemic injury. However, additional research is warranted to confirm the possible acute and chronic effects of silymarin-encapsulated collagen nanoparticles against ischemic stroke.

## Data Availability Statement

All datasets generated for this study are included in the article/Supplementary Material.

## Ethics Statement

The animal study was reviewed and approved by the Animal Ethics Committee of Jamia Hamdard.

## Author Contributions

PR, MS, MA, IA, and SR contributed to the conception and design of the study. PR and SS have contributed to writing sections of the manuscript. All authors have a contribution in manuscript revision, have read and approved the submitted version.

## Conflict of Interest

The authors declare that the research was conducted in the absence of any commercial or financial relationships that could be construed as a potential conflict of interest.
