# Supplementary material for: Collagen Nanoparticle-Mediated Brain Silymarin Delivery: An Approach for Treating Cerebral Ischemia and Reperfusion-Induced Brain Injury
Source: Front Neurosci. 2020 Oct 26;14:538404. doi: 10.3389/fnins.2020.538404 (PMC7649428; doi:10.3389/fnins.2020.538404)
Supplement: Supplementary file 1 [file Data_Sheet_1.zip › Supplementary_Material/Supplemenantary-material.DOCX]

Supplementary Material

# Protocol for the quantification of immunohistopathological slides

# Briefly, an 8-bit RGB image was opened and the image tool in the toolbar was chosen, followed by choosing “Color Deconvolution” as an extension to the “Color” option from the drop down menu (Image > Color > Color Deconvolution ) and stain “H-DAB” was selected. The image got split into three images with the second image titled “Color_2,” being the DAB image was quantified. In the next step, from the toolbar “Set measurement” option under the “Image” tool was chosen and options “Mean gray value” and “Display label” selected. Next Color_2 image window was selected and from the toolbar “Measure” option under the “Analyze” tool was selected. This gave the quantification results in the units of intensity which was then converted into Optical density (O.D) with the following formula:

# O.D = log (Max. intensity / Mean intensity), where Max. intensity = 255 for 8-bit image
