# Supplementary figures and images for: Collagen Nanoparticle-Mediated Brain Silymarin Delivery: An Approach for Treating Cerebral Ischemia and Reperfusion-Induced Brain Injury
Source: Front Neurosci. 2020 Oct 26;14:538404. doi: 10.3389/fnins.2020.538404 (PMC7649428; doi:10.3389/fnins.2020.538404)

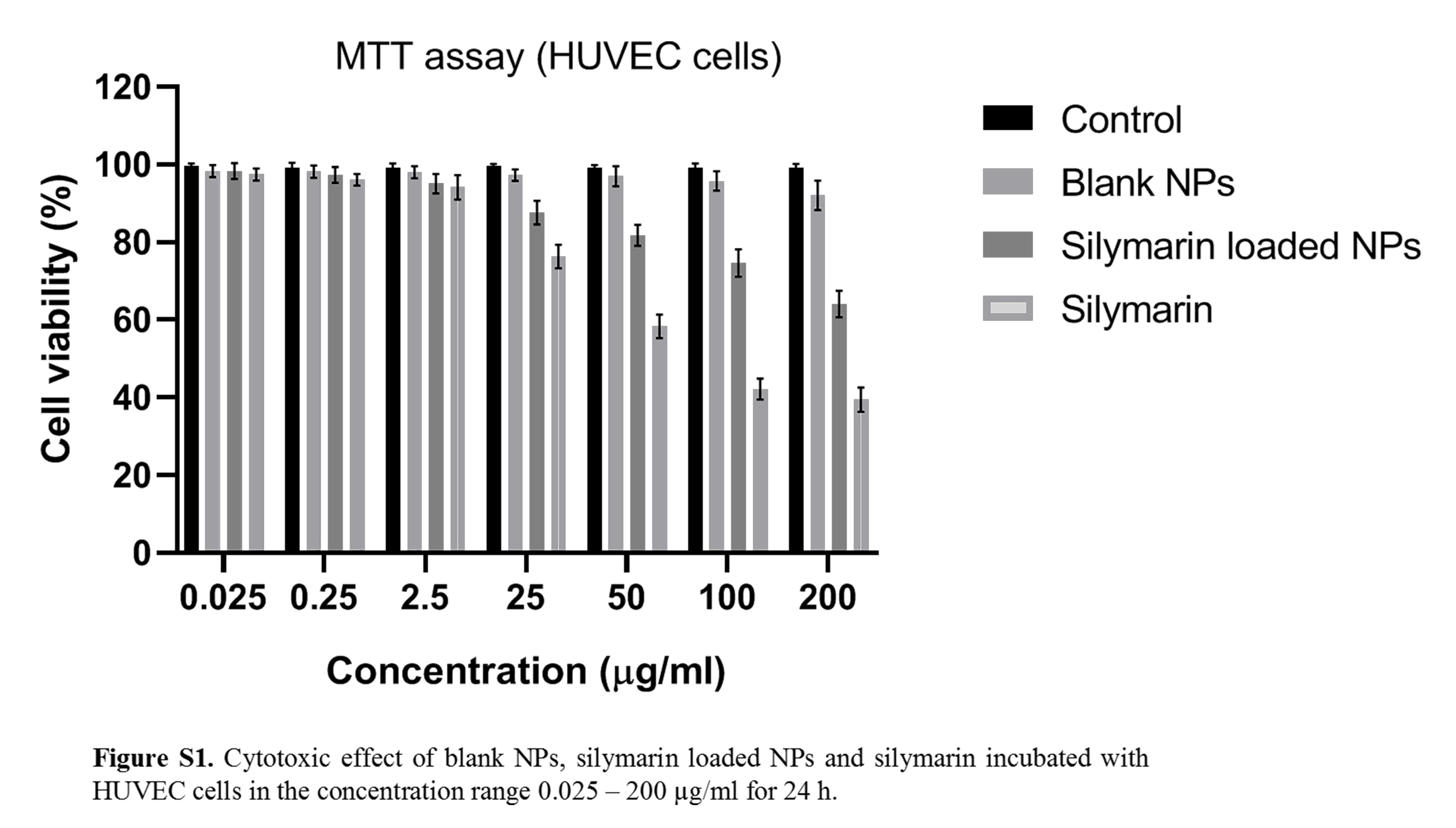

Supplement: Supplementary file 1 [file Data_Sheet_1.zip › Supplementary_Material/Image 1_v1.TIF]
